# Children’s learning preferences for platforms and human informants

**DOI:** 10.1038/s41598-026-56368-x

**Published:** 2026-06-03

**Authors:** Julieta Goldstein, Carolina A. Gattei, Cecilia I. Calero

**Affiliations:** 1 Área de Educación, Escuela de Gobierno, Universidad Torcuato Di Tella, Buenos Aires, Argentina; 2 Laboratorio de Neurociencia, Universidad Torcuato Di Tella, Buenos Aires, Argentina; 3 Instituto de Lingüística - Universidad de Buenos Aires, CONICET, Buenos Aires, Argentina; 4 Consejo Nacional de Investigaciones Científicas y Técnicas (CONICET), Buenos Aires, Argentina; 5 Av. Pres. Figueroa Alcorta 7350, C1428, Autónoma de Buenos Aires, Argentina

**Keywords:** Self-Regulated Learning, Information Seeking, Learning-to-Teach Oneself, Epistemic Evaluations, Education, Psychology, Psychology

## Abstract

As independent learning becomes increasingly important in digital contexts, understanding how children choose and evaluate information sources has become a key aspect of learning research. This study examined children’s reported information seeking behaviors, and how they evaluate and choose sources for learning in hypothetical scenarios. We interviewed 4th- and 5th-graders, inquiring how they would approach learning a novel versus a partially-known concept, and how these choices relate to their epistemic and motivational evaluations of those sources. Children also described what they would do if they felt stuck during the learning process. Overall, considerably more children (73.62%) chose platforms (both printed and digital) rather than human sources. Interestingly, this preference coexisted with higher perceived trustworthiness and anticipated enjoyment associated with human sources. Learning expectations were similar across sources, regardless of concept novelty. When feeling stuck, children who initially chose platforms were more likely to report switching strategies than those who initially chose human sources. These findings contribute to a more nuanced understanding of how children integrate epistemic evaluations, motivational expectations, and perceived uncertainty when making information-seeking decisions in digital learning contexts. Such insights may inform educational practices to better support students’ information-seeking and self-regulated learning, skills that are relevant beyond the classroom in lifelong personal and professional contexts.

**Supplementary Information:**

The online version contains supplementary material available at 10.1038/s41598-026-56368-x.

## Introduction

### Self-regulated learning

As technological advancements continue to reshape an increasingly interconnected world, the ability to learn independently has become an essential competence for continuous education and, widely recognized as a core competence, with considerable implications for both personal and professional success^1,2^. The importance of this ability, commonly referred to as *learning how to learn* and often framed as lifelong learning, is reflected in its inclusion in major global educational frameworks, such as UNESCO^3^, the inter-American Development Bank^4^, and the European Council’s (2006) educational agenda^5^, among others.

The concept of *learning to learn* emphasizes learners’ capacity to direct and monitor their own learning processes, while flexibly adapting strategies across diverse contexts. At its core, it entails *learning to teach oneself*: developing the autonomy and intention to decide what, how, and when to learn. This particular perspective aligns closely with the framework of Self-Regulated Learning (SRL), which refers to the active control learners have over their cognitive, motivational, and behavioral processes to achieve specific learning goals^4,6,7^. SRL involves setting goals, planning, information seeking, monitoring progress, adapting strategies, and engaging in self-assessment, all of which could take place both individually and in social contexts. Central to these processes is metacognition, defined as the ability to reflect on one’s own knowledge and cognitive processes and to regulate them in order to achieve learning goals^8–10^. From this perspective, recognizing what one knows, what one does not know, and when a strategy is ineffective constitutes a metacognitive foundation for adaptive information-seeking. Thus, children’s evaluations of source knowledge, trust, and learning expectations may be understood as metacognitive judgments that guide regulatory decisions during learning. These activities require personal initiative, perseverance, and adaptive skills, collectively fostering autonomy and supporting lifelong learning.

The present study focuses on information seeking as a key component of SRL, defined as the set of cognitive activities undertaken to collect, use, evaluate, and process information^11^, where information broadly refers to any data or insight that may alter an individual’s state of knowledge^12^. As such, information seeking functions as a regulatory mechanism that enables individuals to reduce uncertainty and adapt across educational and social contexts. Yet, despite its central role in *learning to teach oneself*, relatively little is known about how children evaluate and select among different potential sources of information, particularly when comparing traditional human sources with platforms.

### Information-seeking and source evaluation

When learning from others or from external sources, children must evaluate the reliability of the information they encounter. Doing so requires developing the ability to adopt a critical stance toward information, weighing multiple cues to determine whether a source is trustworthy^13^. Research on selective trust and exploratory behavior indicates that children are early and sophisticated evaluators of epistemic credibility.

Asking questions and seeking information are foundational to learning and knowledge acquisition^14,15^. By age three, children can already distinguish between knowledgeable and ignorant informants, showing a preference for learning from the former, both for human agents^16–19^ and computers^20^. By age six, they can adapt their exploratory behavior, increasing inquiry when perceiving that the interlocutor is less knowledgeable, thereby compensating for information gaps^21,22^. These findings highlight children’s early sensitivity to source credibility, which in turn shapes their selective information-seeking. Yet, it remains less clear how this sensitivity translates into choices across different types of sources, particularly as they increasingly navigate both human and digital informants.

Although children are frequently exposed to digital technologies, their understanding of these tools as sources of knowledge remains limited. While children aged 4–6 can readily identify tablets and smartphones as devices for playing games, taking pictures, or watching movies, they are less likely to recognize their potential for work, learning, or communication, and often do not identify them as sources of information^23,24^. More recently, Girouard-Hallam et al.^25^ reported that children’s understanding of the internet develops substantially between ages 7 and 10 with improvements continuing through age 12^26^. This developmental trajectory suggests that middle childhood represents a critical window in which children are still consolidating their understanding of digital technologies as knowledge sources.

At the same time, a meta-analysis on gender differences in attitudes toward technology use found a tendency for men to report more favorable attitudes than women, although these differences are generally small in magnitude^27^. However, findings across individual studies have been mixed, with some reporting the opposite pattern and others no gender differences at all. When considering different components of attitudes, gender differences have decreased over time in affective responses (e.g., enjoyment, anxiety) and self-efficacy (i.e., confidence in using technology), while increasing in belief-related aspects (e.g., perceived usefulness of technology)^27,28^. This evidence is primarily based on samples of secondary school and college students, with no studies focusing on primary-school populations. In addition, effect sizes varied across regions, being larger in North American samples than in Asian and European ones, with no available data from South America. Together, these findings highlight the variability of gender differences across developmental stages and cultural contexts, limiting the extent to which they can be generalized to younger populations.

## Digital versus human informants

Building on this foundation, recent studies have examined how children evaluate information sources in learning contexts. Girouard-Hallam and Danovitch^29^ documented a developmental shift in how children aged 4–12 evaluate human and digital sources: younger children tend to favor familiar human informants, while older children increasingly rely on digital sources such as Google and make greater use of epistemic cues (e.g., perceived knowledge). Notably, children aged 4 and 5 preferred learning factual information from humans over voice-based digital assistants, indicating that their preference for human sources is not limited to text-based information^30^. Relatedly, children this age are also sensitive to the temporal focus of information: the shift is particularly pronounced for present- and future-oriented questions, whereas preferences for past events remain relatively stable, already favoring Google^31^.

Within contemporary digital learning environments, individuals are confronted with vast amounts of information, both accurate and misleading^32,33^. Equipping children with the ability not only to find information, but also to evaluate its credibility, the intentions behind its dissemination, and the reliability of different sources has become essential for fostering critical thinking and lifelong learning abilities^34,35^.

## The present study

In sum, children are sensitive to source credibility and can distinguish between more and less knowledgeable informants across human and digital sources, with older children increasingly relying on digital sources and epistemic cues. Crucially, when seeking information, learners do not always approach the same epistemic conditions. In some cases, they encounter entirely novel concepts for which they have no prior knowledge, whereas in others they engage with topics that are already partially-known. Yet, so far, existing research has not directly examined how learners’ level of prior knowledge shapes how they approach information under different epistemic conditions. We propose that these differences in prior knowledge may influence how learners evaluate and select information sources, as well as the strategies they use when learning.

Extending this emerging literature, the present study investigates how children approach different scenarios that require them to decide how to learn a partially-known or a novel concept and why. Specifically, we examined: (1) the sources that 4th- and 5th-graders report they would select to learn a new concept; (2) whether children’s preferred learning source varied as a function of school grade or gender; (3) whether the choice varies depending on the novelty of the concept to be learned; (4) how these choices relate to their epistemic and motivational evaluations of those sources (i.e., perceived knowledge, trust, expected learning, and enjoyment); and (5) whether children report maintaining or changing their learning strategy when encountering difficulties in a hypothetical learning scenario.

For analytic purposes, children’s responses regarding how they would learn the corresponding concept were grouped into two broad categories: platforms and human sources. In this study, we use the term platforms to refer to non-human information sources, including digital sources (e.g., Google) and printed materials (e.g., books). We contrast these sources with human sources, which comprise individuals from both educational contexts (e.g., teachers) and social contexts (e.g., parents, relatives, or friends). These categories and subcategories were defined to reflect ecologically valid modes of information access in children’s everyday learning environments, capturing both the main interpersonal contexts in which children seek information and the most common forms of non-human information sources.

In particular, the study focused on four evaluative dimensions that might shed light into children’s epistemic and motivational perceptions: (A) perceived knowledge of the source, (B) trust in the source’s response, (C) learning expectations, and (D) expected enjoyment when learning. Dimensions A and B build on prior research about children’s beliefs in omniscience and trust in technological or human sources^31,36^. Dimension C addressed children’s subjective predictions of how easy or difficult it would be to learn about a novel or partially-known concept with a given source^9,37^, capturing expectations about the learning process that are conceptually related to metacognitive perspectives on learning. Finally, dimension D (expected enjoyment) was included to capture the idea, and widely held assumption, that children might prefer sources that are enjoyable or easier to engage with^38^, despite the lack of systematic empirical evidence. Incorporating these dimensions allows us to consider motivational and affective aspects alongside cognitive and trust-related considerations in children’s source selection.

Based on the literature reviewed above, we first hypothesized that children would choose to learn more from platforms when learning about a novel concept (i.e., ‘*Blickets’*) compared to the partially-known (i.e., ‘*Earthquakes’*), reasoning that in the absence of a clear referent, they might default to platforms as a more general or accessible option. We also expected that boys and older children would be more likely to choose to learn from platforms than girls and younger children. Lastly, we predicted that children would perceive both human sources and platforms as more knowledgeable and trustworthy when the concept was partially-known, anticipating lower ratings for the novel concept. While we formulated specific hypotheses regarding source selection and perceived knowledge/trust based on prior literature, predictions concerning anticipated enjoyment, learning expectations, and reported strategy shifts were not specified a priori, as existing research has not directly examined these dimensions within comparable paradigms. These aspects were therefore examined as open research questions.

This study aims to advance our understanding of children’s perceptions of information sources and the reasons underlying their information-seeking strategies. Within the current landscape of information overload, examining how children search for and evaluate reliable information remains an underexplored area, with the potential to inform educational practices that contribute to the development of lifelong learning skills.

## Results

### Children’s prior knowledge of *Blickets* and E*arthquakes* and task engagement

We first examined children’s prior knowledge to verify our design assumptions that *‘Blicket’* represented a novel concept and *‘Earthquakes’* a partially-known concept. Children were asked whether they knew what a *‘Blicket’* is and how an earthquake forms. All children were interviewed about both concepts in a random order. In alignment with our experimental premise, most of the children (97.6%) reported not knowing what a *‘Blicket’* was. Regarding *‘Earthquakes’*, 43.4% of participants reported not knowing how an earthquake forms (no knowledge), while 48.1% provided partial or vague explanations (partial knowledge); for example, children stated “it is a natural disaster” or “it is when the earth shakes.” These explanations did not identify the underlying mechanism of earthquake formation. Finally, 8.5% reported full understanding (accurate knowledge); for example, children said “it forms because of the tectonic plate movement.” Overall, this distribution supports our hypothesis.

Given the complexity of the interview task and to ensure task engagement, we included two control attentional-check questions before continuing with the interview to assess whether children understood and monitored the ongoing task: (a) recalling what they were supposed to learn, and (b) recalling the time they had to present it. Most participants (75.4%) answered both questions correctly, while 21% did not remember when they were expected to present the corresponding concept. A smaller proportion failed to recall either what they were supposed to learn and present (1.2%), or both aspects (i.e., the concept and the time to present it) (2.4%). Children who did not recall either aspect were reminded of the learning task and the time available to complete it.

### Information-seeking: which sources do children turn to?

**Fig. 1:**
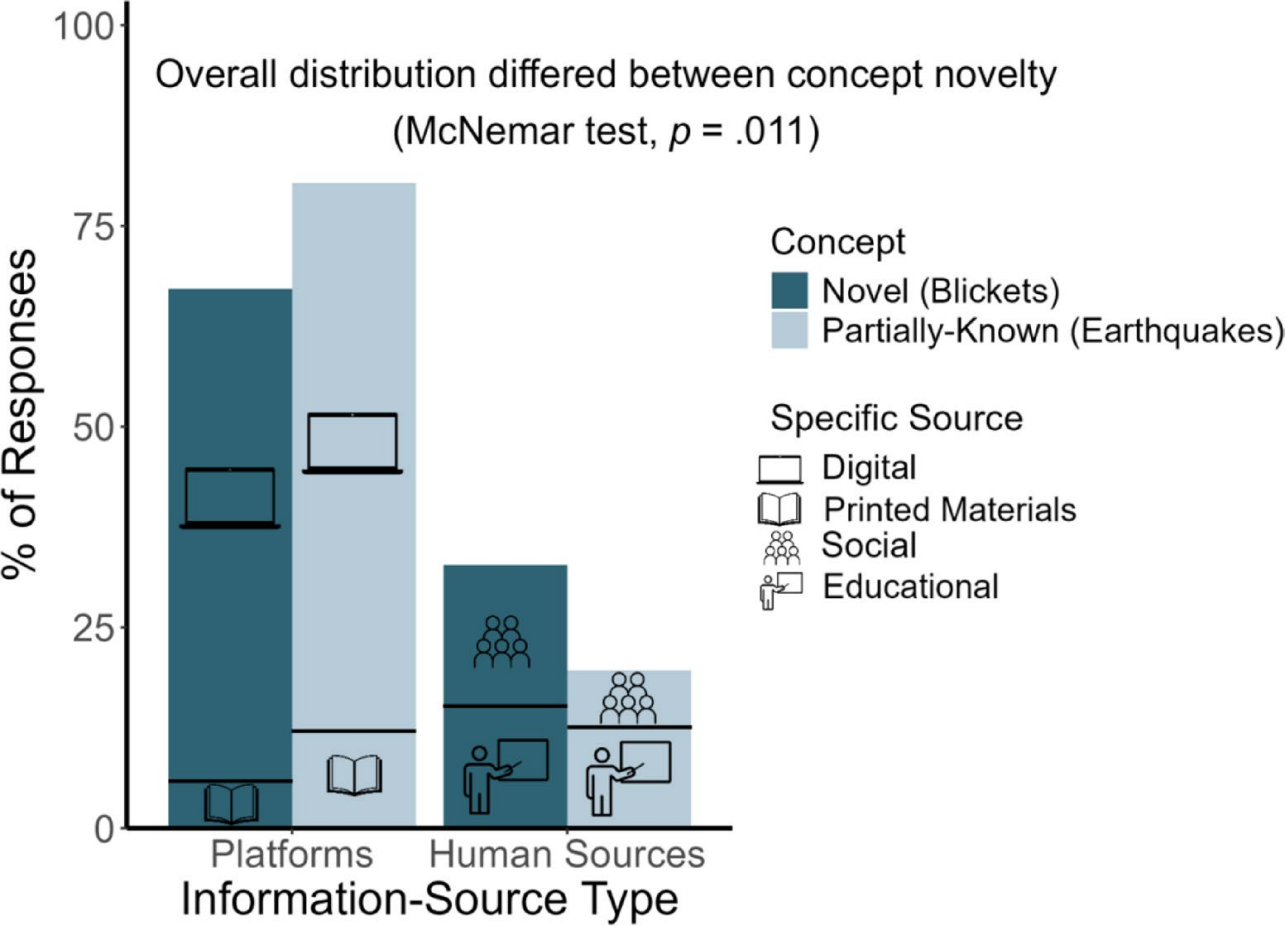
Children’s selection of learning strategies based on concept novelty. Bars show the percentage of children reporting that they would choose platforms versus human sources when presented with either a novel (dark blue) or partially-known (light blue) concept. Icons illustrate specific source types: computer = digital, book = printed materials, group of people = social, teacher = educational,. The overall distribution of source selection differed significantly as a function of concept novelty (*p* < .05).

Children’s source selection was evaluated across the two structured interviews (one per learning concept), yielding a total of 246 responses from 123 participants. Overall, children favored platforms (73.62%) over human sources (26.38%). For both concepts, platforms were selected more often than human sources; however, this difference was larger when the concept was partially-known (‘*Earthquakes’*: 80.47%) than when it was novel (‘*Blickets’*: 66.67%; Fig. 1).

To examine whether children’s choices differed across concepts within participants, we conducted an exact McNemar test, appropriate for paired dichotomous data. Of the 123 participants, 75 consistently chose platforms for both concepts, while 17 consistently chose human sources. The remaining 31 students showed mixed patterns: 23 of them preferred platforms for *‘Earthquakes’*, and human sources for *‘Blickets’*, whereas 8 preferred human sources for *‘Earthquakes’* and platforms for *‘Blickets.’* The exact McNemar test indicated a significant shift in source selection (*p* = .011), with children being significantly less likely to choose platforms for the novel concept than when it was partially-known. The odds ratio was 2.875, 95% CI [1.24, 7.23], which indicated that children who chose platforms for *‘Earthquakes’* were nearly three times more likely to select human sources for *‘Blickets’* than those showing the opposite pattern (see Fig. 1).

Taken together, these results indicate that children more frequently chose platforms than human sources across conditions. However, this pattern was attenuated (but not reversed) when the concept was novel, as children were less likely to report choosing platforms for the novel concept than for the partially-known one. Thus, contrary to our first hypothesis, children showed a stronger tendency to choose platforms when the concept was partially-known rather than when it was entirely novel, suggesting that familiarity may be associated with greater reliance on digital sources.

#### Note

*Icons from The Noun Project (authors: Alzam*,* Gan Khoon Lay*,* Lailistudio*,* Noodle Research).*

To further analyse whether school grade or gender predicted children’s choices, we conducted a generalized linear mixed-effect model including concept novelty (novel vs. partially-known), gender, and school grade as fixed effects, with random intercepts for participants. Because we had no a priori hypotheses regarding interactions among these variables, interaction terms were not included in the model. The effect of novelty remained significant (β = 1.09, SE = 0.395, z = 2.76, *p* = .006), indicating that children were relatively more likely to choose human sources when the concept was novel than when it was partially-known, suggesting a relative shift toward human sources when engaging with a novel concept. Neither gender nor school grade reached significance (*p’s* > 0.10), suggesting that this preference was consistent across groups. Thus, contrary to our hypothesis, neither gender nor school grade predicted children’s source choices.

A closer examination revealed that children’s platform choices were predominantly digital (88.24%), with a much smaller proportion corresponding to printed materials (11.76%). A binomial test confirmed that the proportion of digital choices was significantly greater than chance (95% *CI* [0.83, 0.92], *p* < .001). In contrast, human sources were more evenly distributed between educational (53.73%) and social contexts (46.27%), with the proportion of educational choices not differing significantly from chance (95% *CI* [0.41, 0.66], *p* = .625) (Fig. 1).

### Behind the choices: How source selections relate to epistemic and motivational evaluations

We further examined four evaluative dimensions of the selected information source when learning about *‘Blickets’* or *‘Earthquakes’*: (A) Perceived knowledge of the source, (B) Trust in the source’s response, (C) Learning Expectations, and (D) Expected enjoyment when learning. Participants rated each of these dimensions using a 1–7 Likert Scale, where 1 meant ‘*very little*’ and 7 meant ‘*a lot.*’ Therefore, higher values in the scale indicated more favorable evaluations.

Because these ratings were ordinal, we analysed each dimension using Cumulative Link Mixed Models (CLMM). In each model, the dependent variable was the ordinal rating for the respective evaluative dimension. Fixed effects included the chosen information source (platforms or human sources), concept novelty (*‘Blickets’*: novel, ‘*Earthquakes’*: partially-known), and their interaction. Separate models were estimated for each dimension. Random intercepts for each participant were included to account for the non-independence of repeated observations between individuals.

1. Perceived knowledge of the source.

Children rated how knowledgeable they believed the chosen source was about *‘Blickets’* or *‘Earthquakes.’* A Cumulative Link Mixed Model (CLMM) revealed a significant interaction between information source and concept novelty (*β* = 1.35, *SE* = 0.65, *z* = 2.10, *p* = .036, *N*_*Blickets*_ = 124 and *N*_*Earthquakes*_ = 127). This interaction indicates that the effect of novelty differed depending on the selected source. Follow-up pairwise comparisons showed that when human sources were selected, perceived knowledge was lower for the novel concept (‘Blickets’) than for the partially known concept (‘Earthquakes’) (*p* = .003). In contrast, no significant difference between concepts was found when platforms were selected (*p* = .288). In addition, a main effect of source showed that children choosing human sources rated their informants as less knowledgeable than those who chose platforms (*β* = -1.16, *SE* = 0.42, *z* = -2.74, *p* = .006).

**Fig. 2:**
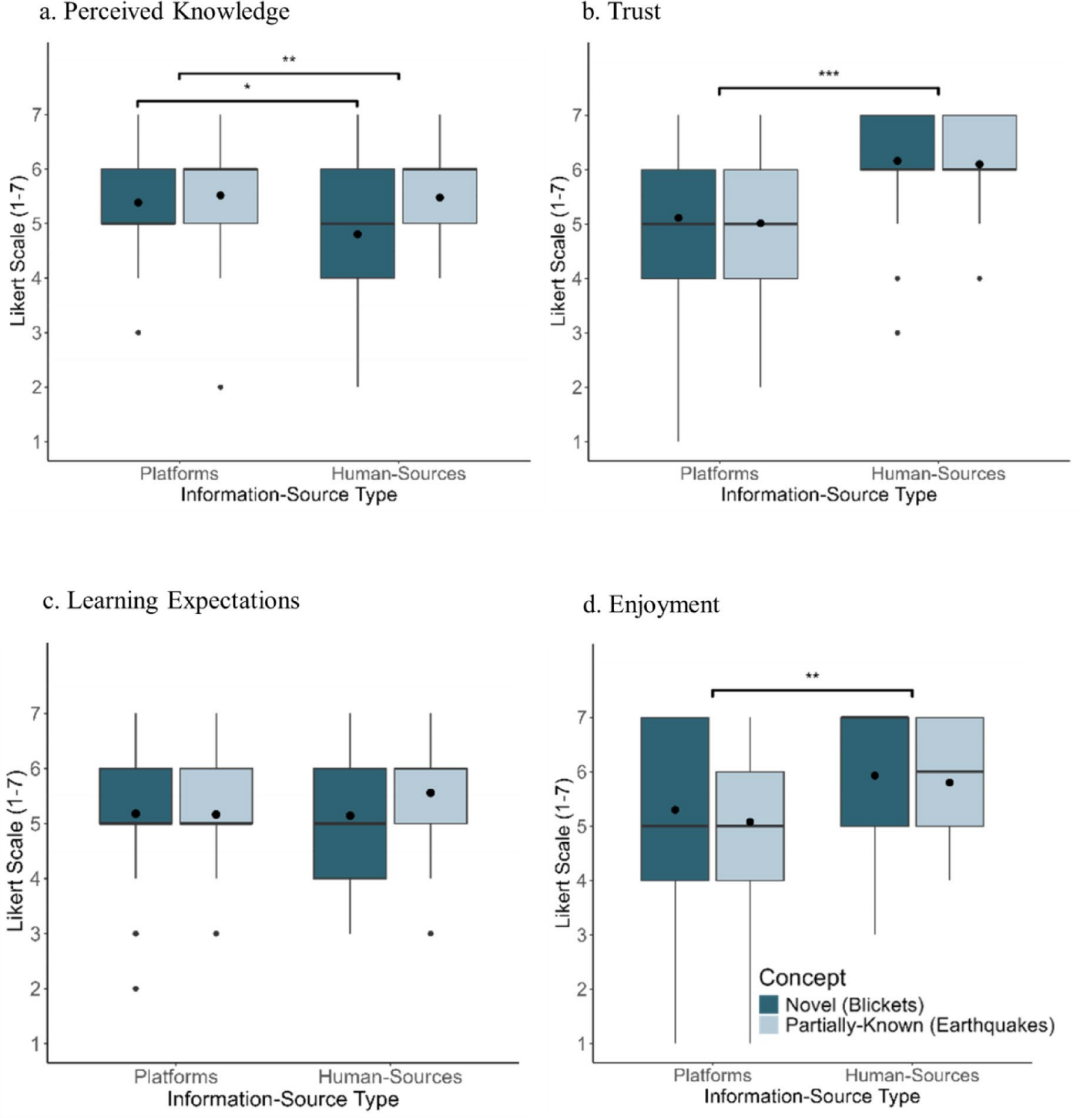
Children’s evaluations of learning sources across four dimensions. (**a**) Perceived knowledge of the source; (**b**) Trust in the source’s response; (**c**) Learning expectations, and (d) Expected enjoyment when learning. Ratings were provided on a 7-point Likert scale (1 = ‘very little’, 7 = ‘a lot’). Tukey boxplots show the distribution of ratings for novel (*‘Blickets’*, dark blue) and partially-known (*‘Earthquakes’*, light blue) concepts. Boxes represent the interquartile range (IQR), with the median shown as the center line, whiskers extending to 1.5 × IQR, and black dots inside the box indicating mean values. Asterisks denote statistically significant differences. Only significant differences were marked. *p < .05, **p < .01, ***p < .001.

Descriptively, children who chose platforms assigned higher ratings to their informants (*M* = 5.46, *SE* = 0.07) than those who chose human sources (*M* = 5.06, *SE* = 0.14). Lower ratings for human sources were particularly observed when evaluating ‘Blickets’ (*M* = 4.805, *SE* = 0.175), whereas ratings of human knowledge about ‘Earthquakes’ (*M* = 5.48, *SE* = 0.20) were comparable to those given to platforms (See Fig. 2a). Within platforms, perceived knowledge ratings were comparable across concepts (‘Blickets’: *M* = 5.39, *SE* = 0.10; ‘Earthquakes’: *M* = 5.52, *SE* = 0.09).

2. Trust in the source’s response.

Children rated how trustworthy they perceived the chosen source’s response to be about ‘Blickets’ or ‘Earthquakes.’ A CLMM revealed a significant main effect of information source (*β* = 2.39, *SE* = 0.56, *z* = 4.26, *p* < .001). Specifically, children who chose human sources rated their informants’ responses as more trustworthy (*M* = 6.137, *SE* = .13) than those who chose platforms (*M* = 5.059, *SE* = 0.10) (*N*_*Blickets*_ = 94 and *N*_*Earthquakes*_ = 95) (see Fig. 2b). There was no significant main effect of concept (*p* = .56), nor an interaction between information source and concept (*p* = .76). Thus, contrary to our hypothesis, perceived trustworthiness did not vary as a function of concept novelty.

3. Learning expectations.

Children rated how much they expected to learn from the chosen source about ‘Blickets’ or ‘Earthquakes.’ A CLMM analysis revealed no significant main effects of concept novelty (*p* = .97) or information source (*p* = .83), nor a significant interaction between the two (*p* = .28). The ratings of expected learning were similar for platforms (*M* = 5.17, *SE* = 0.07) and human sources (*M* = 5.30, *SE* = 0.15) (*N*_*Blickets*_ = 126, *N*_*Earthquakes*_ = 128) (see Fig. 2c). These results suggest that children perceived learning from platforms and human sources as comparably advantageous, regardless of the concept.

4. Expected enjoyment when learning.

Children rated how much they would enjoy learning with the chosen source about *‘Blickets’* or *‘Earthquakes.’* A CLMM analysis showed a significant main effect of information source (*β* = 1.31, *SE* = 0.47, *z* = 2.78, *p* = .005). Specifically, children who chose human sources assigned higher enjoyment ratings (*M* = 5.88, *SE* = 0.15) than those selecting platforms (*M* = 5.18, *SE* = 0.11) (*N*_*Blickets*_ = 126 and *N*_*Earthquakes*_ = 127). No significant main effect of concept novelty (*p* = .34) nor an interaction between source and concept (*p* = .85) was found (see Fig. 2d).

## Navigating obstacles: Children’s strategies when feeling stuck

After completing the first part of the interview for each concept, children were asked what they would do if they got stuck during the learning process. Responses were categorized into three groups: (A) No Change: children who maintained the same strategy as their initial choice throughout the interview; (B) Change Within Modality: children who adjusted their approach within the same modality (e.g., switching from one platform to another or from one human source to another); and (C) Change Across Modalities: children who shifted to a different modality compared to their initial choice (e.g., switching from a platform to a human source, from a human source to a platform, or adopting an alternative approach such as taking a break or summarizing the content).

To examine patterns of strategy change, we conducted a Cumulative Link Mixed Model (CLMM) on 254 responses from 131 participants. The dependent variable was strategy change (No Change, Change Within Modality, or Change Across Modalities). Fixed effects included concept novelty (‘*Blickets’* vs. ‘*Earthquakes*’) and the initially selected source (human source vs. platform), as well as their interaction. A random intercept for participants was included to account for repeated measures.

**Fig. 3:**
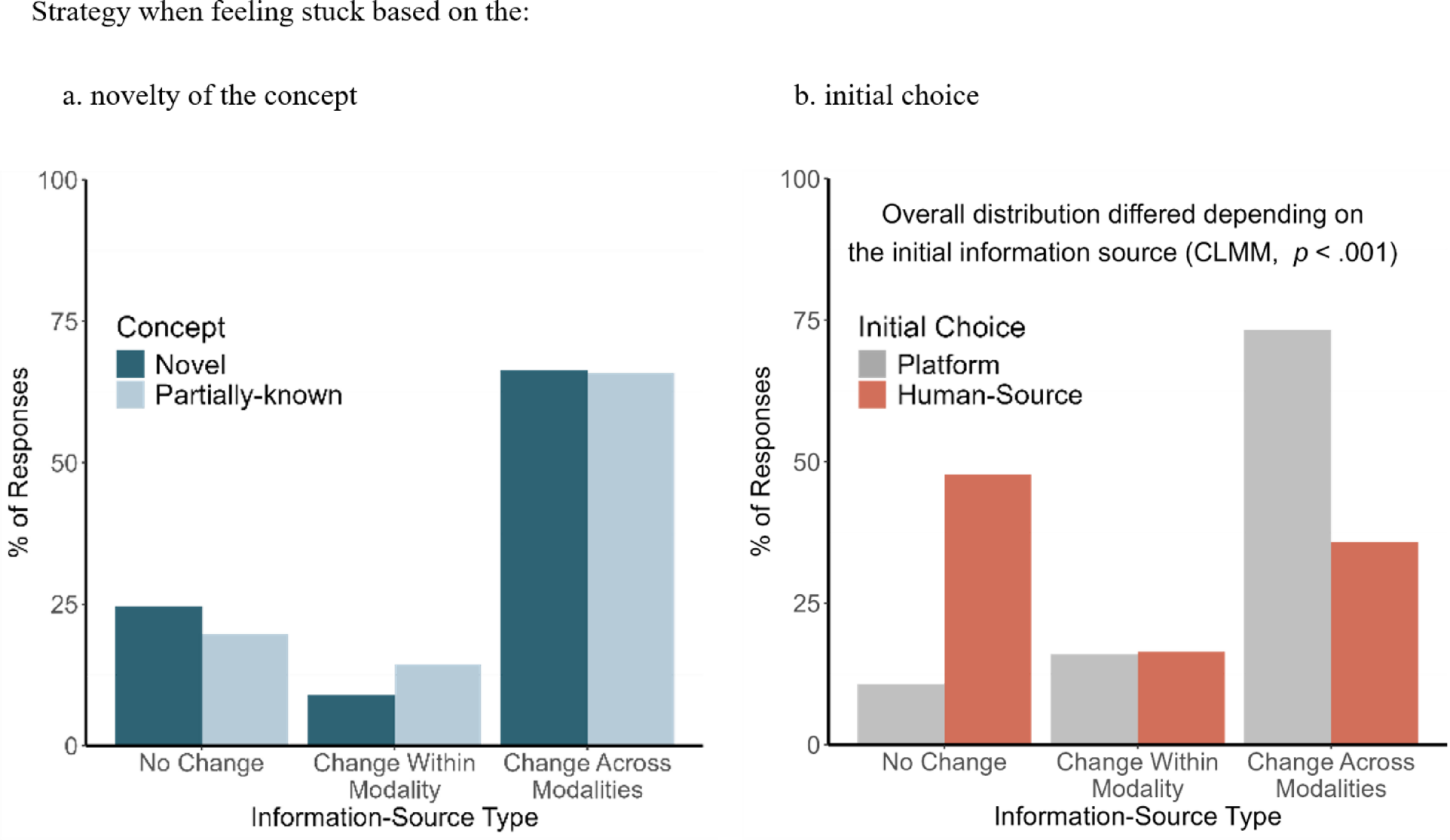
Children’s strategies when feeling stuck. Bars show the percentage of responses for each strategy (No Change, Change Within Modality, and Change Across Modalities) based on: (**a**) whether the concept to be learned is novel (dark blue) or partially-known (light blue); and (**b**) whether the children’s initial learning strategy was a platform (gray) or a human source (orange). The overall distribution of strategies differed depending on the initial information source (CLMM, *p* < .001).

The analysis revealed a significant main effect of the initially selected source: children who initially chose to learn with platforms were significantly more likely to report more extensive strategy changes than those who chose human sources (*β* = 2.493, *SE* = 0.57, *z* = 4.34, *p* < .001). There was no main effect of concept novelty (*p* = .40), nor an interaction effect between the selected source and the concept (*p* = .68) (see Fig. 3). Follow-up analyses using estimated marginal means indicated that, when children reported that they would select platforms, they were significantly more likely to report changing across modalities than either remaining within the same modality or not changing at all (*p’s* < 0.001). No significant differences between strategies were observed when children reported that they would select human sources (all *p’s* > 0.28).

Descriptively, most responses involved switching across modalities, (63.39%), whereas changes within the same modality were less frequent, (16.14%), and 20.47% of responses indicated no change in strategy. When children initially chose platforms, most strategy changes involved switching across modalities, (73.26%), whereas changes within the same modality were less frequent, (16.04%), and only 10.70% reported no change in strategy. In contrast, children who initially chose human sources were more likely to maintain the same strategy, (47.76%).

## Discussion

The present study examined 4th- and 5th-graders’ information-seeking behaviors in hypothetical scenarios. Children were asked how they would learn either a novel or a partially-known concept, how their source selections relate to epistemic and motivational evaluations, and the strategies they would use if they felt stuck. These components capture key aspects involved in SRL, including the selection of learning resources, the evaluation of their usefulness, and the regulation of strategies when difficulties arise.

While our analytic grouping contrasted human versus non-human sources, the observed preference largely reflects children’s inclination toward digital sources rather than print-based materials. This distinction is important, as digital and printed sources differ in terms of interactivity, accessibility, and perceived immediacy.

Most participants indicated that they would choose to learn from platforms rather than from human sources. Given that the platform category consisted predominantly of digital sources (88.24%), this pattern aligns with prior research showing that children increasingly attribute epistemic competence to technological informants for factual information^29,31,39^. Previous studies have documented that children view tools such as Google as capable of providing broad and efficient access to information, particularly for fact-based queries^31,39^. Our findings extend this literature by showing that this preference persists when children consider how they would approach learning in hypothetical scenarios within our protocol.

Children’s preference for platforms over human sources did not differ significantly by gender. Although prior research has documented small gender differences in attitudes toward technology use, these effects are typically modest and vary across contexts and attitudinal components^27,28^. Moreover, existing evidence is largely based on older populations and different cultural settings, limiting its generalizability to primary-school children. Given the lack of research in this specific age group, it remains unclear whether similar gender differences would be expected.

At the same time, no significant differences were observed as a function of school grade. This absence of grade-level differences should be interpreted cautiously. Because exact ages were not collected due to ethical constraints, and because of the overlap in ages between grades, our analyses may have lacked the precision necessary to detect subtle developmental shifts across middle childhood.

When examining the evaluative dimensions associated with children’s choices, a dissociation emerged between perceived epistemic competence and source selection. With respect to perceived epistemic competence, an interaction emerged between information source and concept novelty. While children’s evaluations of human sources were sensitive to novelty, being lower for the novel concept, their perceptions of platform knowledge were relatively similar across both novel and partially-known concepts. In fact, ratings of human sources in the partially-known (earthquake) condition were comparable to those assigned to platforms, whereas lower ratings for human sources were primarily observed in the novel (Blicket) condition. This pattern suggests that the effect of novelty on epistemic evaluations was contingent on the information source type considered (Human or Platforms). This finding aligns with prior research indicating that children often attribute broad epistemic authority to technological tools, particularly for factual information^31,39^, and raises the possibility that children may perceive digital platforms as possessing a form of domain-general epistemic authority. From a cognitive perspective, this pattern may also reflect egocentric anchoring processes: children’s own unfamiliarity with *‘Blickets’* may have led them to infer that human sources would be similarly uninformed^40,41^, consistent with a more context-dependent evaluation of human knowledge.

Paradoxically, despite platforms being rated as more knowledgeable, children turned more frequently to human sources when confronted with novelty (relative to the partially-known concept). This suggests that perceived knowledge alone does not fully determine information-seeking decisions.

In addition to knowledge attributions, children who chose human sources rated their informants as more trustworthy and expected greater enjoyment than children who chose platforms, whereas learning expectations did not differ between sources. Notably, these evaluative ratings did not directly mirror children’s source selections. Although children choosing human sources perceived informants as more trustworthy and more enjoyable, platforms were still selected more frequently overall. This dissociation between epistemic evaluations and imagined source selection suggests that children’s information-seeking decisions may not be guided solely by their explicit judgments about a source’s knowledge or trustworthiness. Developmental research has similarly emphasized that adopting a critical stance toward information involves weighing multiple cues and contextual factors when evaluating sources^13^. One possible explanation is that factors beyond epistemic evaluations may influence children’s choices. For instance, availability may be one such factor. Girouard-Hallam and Danovitch^39^ found that children view Google as faster than a person when seeking factual information, which may influence their choices, particularly given that this study’s hypothetical scenario framed learning within a three-hour time constraint. In addition, because data were collected in school settings, some participants may have interpreted the task as school-related, potentially shaping their responses. Although availability was not systematically assessed in the present study, some qualitative evidence from participants’ justifications is consistent with this possibility. Children who reported low expectations of learning or enjoyment (ratings of 3 or below) were asked to justify their choices. Among those selecting platforms, approximately half referred to time constraints or ease of access. In contrast, among those selecting human sources with similarly low expectations, about half cited the informant’s preparation or expertise. However, these observations should be interpreted with caution, as they were not derived from systematic analyses. Further systematic research should be conducted to examine how availability, as well as related contextual factors such as time constraints and task framing, may shape children’s reasoning when choosing between information sources.

Children’s strategies when feeling stuck offer further insights into how information-seeking may unfold within SRL contexts. Participants who initially selected platforms for these hypothetical scenarios were significantly more likely to report switching strategies compared to those who imagined relying on human sources, regardless of whether the concept was novel or partially-known. From a metacognitive perspective, strategy switching may reflect a regulatory response to perceived difficulty, involving monitoring and adaptive help-seeking^8,9,42^. One possible explanation is that children recognize that humans can provide interactive, adaptive, and affectively supportive feedback that platforms could not readily offer at the time of data collection, when conversational AI tools were not yet widely used by children of this age group (for instance, Chat GPT was released in November 2022, after data collection for our study had already begun). However, the present data do not fully allow us to disentangle whether the greater likelihood of switching among children who initially selected platforms is driven by a tendency to move toward human informants, or reflects a more general propensity to change strategies.

At the same time, existing literature suggests that children’s understanding of how the internet functions remains under development through age 12^26^, and that they often struggle to evaluate the accuracy of online content^43^, formulate queries successfully^44^, or distinguish advertisements from informational content^45^. Within this context, the greater likelihood of switching away from platforms may reflect emerging awareness of the limitations of digital search or difficulties in navigating online environments.

From an educational perspective, these results highlight the importance of considering not only children’s initial preferences for sources, but also how they would adapt their strategies when encountering obstacles. Together with prior evidence, the present results highlight the potential value of interventions aimed at strengthening children’s ability to critically select and evaluate information sources.

Beyond the task constraint regarding time and school setting, several limitations should be noted. First, the study relied on children’s reported preferences in hypothetical learning scenarios rather than on observed behavior or actual learning performance. Consequently, the findings reflect children’s stated intentions and anticipated strategies, which may not fully correspond to the decisions they would make in real-world contexts. Self-report measures are inherently limited in that children responding to hypothetical scenarios may indicate what they believe is expected or reasonable, rather than what they would actually choose under situational constraints such as time pressure, effort, or accessibility. At the same time, the hypothetical format allowed us to examine children’s spontaneous repertoires of information sources without constraining their responses to predefined alternatives. This approach provides a useful foundation for subsequent research examining actual source selection and learning behavior within structured tasks.

A further limitation concerns the sequential nature of our design. Children first made their source selection in an open-ended format and only afterwards provided evaluations of the selected sources. This design choice was intentional, as it aimed to capture children’s spontaneous source preferences without constraining their responses to predefined categories, and avoiding the potential priming effects that could arise if evaluations were elicited prior to choice. However, as a consequence, we cannot determine whether epistemic and motivational beliefs strictly preceded and influenced source selection, or if children’s ratings were partially influenced by the choice itself (e.g., as a form of post-hoc justification). Furthermore, this sequential structure precludes within-subject comparisons between selected and non-selected sources. This limits the possibility of testing mediation or directional relationships between these variables, and may also account for some of the observed discrepancies between children’s choices and their ratings.

Another limitation concerns the classification of earthquakes as a partially-known concept. Children were asked whether they knew how earthquakes form, which specifically assessed their ability to explain the mechanism underlying the phenomenon. Some children who reported not knowing how earthquakes form may nevertheless have been familiar with earthquakes at a more general level (e.g., recognizing them as natural disasters). Because follow-up explanations were not requested when children responded that they did not know, we cannot determine the extent to which these responses reflected a complete lack of knowledge or difficulty articulating the causal mechanism. Therefore, the classification of earthquakes as a partially-known concept should be interpreted with caution.

In addition, it is important to note that only a subset of children (26.38%) showed differentiated source choices across both concepts (*Blickets* or *Earthquakes*). This pattern suggests that the observed effect of novelty may be driven by a subgroup of participants and should therefore be interpreted with caution.

Future research could address these limitations by (a) extending the time frame of the task and disentangling it from school contexts, (b) examining how children’s stated preferences translate into actual behavior choices and learning outcomes, (c) investigating how children engage with and regulate platform use in naturalistic settings, and (d) incorporating within-subject designs in which children evaluate multiple potential sources prior to making a selection, in order to better disentangle the relationship between evaluative judgments and information-seeking behavior, while also considering how such designs may influence children’s responses through comparison or consistency processes.

Overall, this study represents an initial step toward understanding which information sources children choose, the factors associated with these choices, and how they evaluate those sources. By documenting the interplay between perceived knowledge, trust, motivational expectations, and adaptive strategy use, the findings contribute to a growing understanding of how children navigate learning decisions in environments characterized by unprecedented access to diverse and competing sources of information. These insights provide a foundation for future research that directly links children’s perceptions to their actual learning behaviors and outcomes, an essential step for informing educational practices aimed at fostering critical information-seeking and supporting effective Self-Regulated Learning.

### Methods

#### Participants

A total of 134 fourth- and fifth-grade students were recruited from three schools in the Buenos Aires Metropolitan Area, Argentina (predominantly Hispanic-Latino and Caucasian). Due to ethical committee constraints, participants’ exact ages were not collected; typical age ranges were 8–10 years for fourth graders and 9–11 years for fifth graders. Three participants were excluded due to attentional challenges that compromised their full engagement during the study, resulting in a final sample of 131 children (54.2% girls, 45.8% boys; 46.6% 4th-graders, and 53.4% 5th graders). Additionally, for six children, one interview each was excluded due to experimenter errors (four from the *novel concept* condition and two from the *partially-known condition*).

The study received approval from an Ethical Committee: Comité de Ética Para la Investigación Científica y Tecnológica de la Universidad Abierta Interamericana (CEICyT – UAI), Dictamen N° 1098 and was conducted in accordance with relevant guidelines and regulation, particularly with ethical principles established for the care and respect of children’s rights - United Nations Convention on the Rights of the Child, Law No. 26,061. All data remained confidential and anonymized, with each participant assigned a unique ID number. Parents or legal guardians of all participants provided signed informed consent for their children’s participation and all children provided oral assent.

A total of 126 responses were collected for the *‘Blicket’* interview and 128 for the *‘Earthquakes’* interview. Sample sizes varied across analyses for the following reasons: (i) in the Selected Source Analysis, only children who provided a choice of a platform or human source (see Design and Procedure) in both interviews were included, in order to allow repeated measures comparisons (participants discarded from one interview or giving other types of answers in either session were excluded); (ii) in the Trustworthiness Analysis, one school was excluded due to a change in the wording of this question and (iii) all “*I do not know*” responses were excluded from the specific analyses involving those questions.

### Design and procedure

A semi-structured interview with pre-established questions was designed to examine children’s information-seeking behaviors. Each child completed two interviews, one on a novel concept, ‘*Blicket*’, and one on a partially-known concept, ‘*Earthquakes*’, presented in randomized order. These interviews were administered through one-on-one interviews, and all sessions were audio-recorded. Each interview lasted an average of 6.18 min (range = 3–15 min). Data were collected between November 2022 and November 2023, with the two interviews administered 1 to 5 weeks apart.

Each child was individually taken to a designated space (classroom or office, depending on each school’s availability) to meet the experimenter. We ensured that no computers, books, or other individuals were present in the space to prevent any potential biases in their responses. Interviews were assessed orally to minimize written limitations. At the start of the interview, children were asked to imagine they should prepare a presentation in three hours for their science course that same afternoon. In the presentation, they would have to explain either (i) *What is a ‘Blicket’* or (ii) *How an earthquake is formed*.

1. A *‘Blicket’* is a non-existing word, frequently used in cognitive science^46^. Therefore, a ‘*Blicket’* represented the *Novel Concept*.
2. On the other hand, students this age in Buenos Aires, Argentina, have heard very little about earthquakes, as the region is characterized by very low seismic activity^47^ and earthquakes are not included in the 4th- and 5th-grade curriculum. Most children had a general idea of what an earthquake is, but they did not know how it forms or could barely grasp its meaning. Therefore, ‘*Earthquakes’* represented the *partially-known concept*.

After introducing the statement, we checked their previous knowledge of each concept by asking whether they knew what a ‘Blicket’ is or how earthquakes form (e.g., “Do you know what a Blicket is?”; “Do you know how earthquakes form?”). We used these responses to validate our assumption that ‘Blicket’ would function as a novel concept and ‘Earthquakes’ as a partially-known one. When children reported some degree of knowledge (e.g., “yes” or “more or less”), they were asked to briefly explain their response, and these explanations were used for coding (see Data Coding). Additionally, we asked them two control questions to check their attention to the statement: We inquired if they remembered when they had to present at school and what concept they were expected to present.

Following the attentional questions, children were first asked an open-ended question: “*How would you learn [the corresponding concept]*?” Children were free to indicate any source they would use to learn the concept. Their responses were later coded into predefined categories (platforms vs. human sources). They were then asked several questions to explore the main dimensions underlying their choices on how to learn each of the two concepts: (A) “*How much do you think [the chosen source] knows about [the corresponding concept]?”*, (B) “*How much do you trust in [the chosen source’s] response when asking about [the corresponding concept]?”*, (C) “*How much do you expect to learn with [the chosen source] about [the corresponding concept]?”*, and (D) “*How much do you expect to enjoy the learning process?”* (i.e., always in reference to their selected source, not a generic one). Children rated each dimension on a 7-point Likert Scale, where 1 indicated ‘*very little*’ and 7 indicated ‘*a lot.*’ Higher values therefore indicated more favorable evaluations. The scale was presented on a printing material, with 1 shown in light blue and progressively darkening to dark blue at 7.

Lastly, children were asked: “*What would you do if you got stuck during the learning process?”* The change in their learning strategy was analysed.

Based on their responses, children were grouped into two categories: those who chose to learn from platforms and those who chose human sources for each concept. Platforms included children who chose to learn using digital sources (e.g., Google) or printed materials (e.g., books). Human sources included those who chose to learn from people, either within the educational setting (e.g., teachers) or from social, non-educational contexts (e.g., parents, relatives, or friends). Only two participants, one in each interview, provided responses that did not fit to either category and were excluded from the analysis.

### Data coding

Children’s responses to their prior knowledge, attentional checks and open-ended justifications for their ratings were classified using a deductive coding scheme with predefined categories. A glossary was developed in advance to define each category and specify how children’s responses to the interviews would be classified.

Ordinal responses included attentional checks, which were coded as 0 (could not retrieve the answer) or 1 (correctly retrieved the answer) and prior knowledge, which was coded into three categories: 0 = no knowledge, when children explicitly reported not knowing; 1 = limited or partial knowledge, when children provided vague or partially incorrect explanations; and 2 = accurate knowledge, when children demonstrated a more precise understanding (e.g., referring to tectonic plate movement). When children reported some degree of knowledge (e.g., “yes” or “more or less”), they were asked to briefly explain their answer, and their explanations were used to determine the coding category. When children reported that they did not know, no follow-up explanation was requested. Therefore, although these responses were coded as “no knowledge”, it remains possible that some children possessed minimal familiarity with the concept of earthquakes without being able to explain their origin. This should be considered when interpreting the classification of earthquakes as a partially-known concept.

Categorical responses included the selected source (digital sources, printed material, educational human source and social human source), the strategies children would use if they felt stuck, which coders recoded as same or another digital source, printed material, educational or social human source, or other strategies such as taking a break or making a summary. In addition, children who rated learning expectations or expected enjoyment at 3 or below, were asked to justify their reason for selecting the source. These responses were categorized into predefined categories such as *Time* (e.g., “because I only have three hours” or “because it is faster”), *Ease of Access* (e.g., “because it’s easy, I just ask Google and it tells me”), and *Preparation/Expertise* (e.g., “because the teacher should know, she studied to teach about it”).

During the interviews, the experimenter coded children’s responses in real time, using the predefined categories. Since the interviews were audio-recorded, the initial coding could be verified afterward. One coder coded the entire dataset, and a second coder independently coded 57.25% of the data.

Inter-rater reliability was assessed using Cohen’s Kappa^48^ in order to estimate the coding concordance. This analysis should be conducted separately for ordinal and nominal data. For the ordinal data, a total of 295 responses (those corresponding to the control and attentional questions) were included and assessed using squared weights, which account for the ordinal nature of the responses and provide a more refined measure of agreement. The Kappa value obtained in the coding of the ordinal responses was 0.94, which is considered an ‘almost perfect’ agreement^49^. Likert-scale questions concerning the epistemic and motivational evaluations of the source were not included in this analysis, as they did not require interpretation.

Likewise, inter-rater reliability for the nominal responses was evaluated using unweighted Cohen’s Kappa, which treats all disagreements equally and is suitable for nominal data where the categories have no intrinsic ordering. This analysis included 338 responses, with a Kappa value of 0.86, reflecting a strong agreement between the two coders in their classification of nominal responses.

### Data analysis

Data analysis was conducted using *R* software^50^. The following packages were utilized: irr^51^, pwr^52^, emmeans^53^, rcompanion^54^, ordinal^55^, and lme4^56^.

To assess within-participant differences in source selection across the two learning concepts (novel vs. partially-known), an Exact McNemar test was conducted, as responses were paired and categorical. To further explore potential effects of children’s gender and school grade on source selection, a binomial Generalized Linear Mixed Model (GLMM) with random intercepts for participants was fitted, allowing the inclusion of repeated measures and participant-level covariates. In addition, Cumulative Link Mixed Models (CLMM) were employed for the analysis of Likert scale ratings and the evaluation of children’s reported strategies when feeling stuck, as these models are specifically suited for ordinal outcomes and can incorporate random effects to control for inter-individual variability. None of the statistical models assumed normal distributions, as they are designed for either binary, categorical, or ordinal data. All tests were two-tailed, with a significance level of α = 0.05.

## Supplementary Information

Below is the link to the electronic supplementary material.

Supplementary Material 1

Supplementary Material 2

## Data Availability

The datasets generated and analysed during the current study are not publicly available due to ethical considerations, as they include children’s information. However, they may be made available from the corresponding author on reasonable request.

## Code availability

The underlying code for this study is not publicly available but may be made available to qualified researchers on reasonable request from the corresponding author.
