# Supplementary material for: Children’s learning preferences for platforms and human informants
Source: Sci Rep. 2026 Jun 3;16:25371. doi: 10.1038/s41598-026-56368-x (PMC13473612; doi:10.1038/s41598-026-56368-x)
Supplement: Supplementary file 2 [file 41598_2026_56368_MOESM2_ESM.pdf]

ID:

EARTHQUAKES

Interview No.:

Start time:

Imagine that at school you are asked to give a presentation this afternoon explaining “where earthquakes come from” for your Natural Sciences class. Imagine that you have to deliver the presentation this afternoon in 3 hours, and during this time you need to learn “where earthquakes come from”.

Control questions:

- Do you know where earthquakes come from?
- When do you have to give the presentation?
- What do you need to learn in order to prepare the presentation?

Then, how would you learn about earthquakes in order to prepare the presentation?

If the participant gives more than one answer:

Record all responses and ask: “And if you had to choose just one option?”

That answer is the one used for the rest of the interview.

If the participant does not know, prompt again:

1st prompt: “Try to imagine it, think about it.”

2nd prompt: Repeat the story.

3rd prompt: “And if you had to guess?”

If the participant still does not know, end the interview.

If the participant gives an answer: Record “Don’t know,” the number of the prompt at which the participant responded, and the chosen source.

ID:

1. How much does … [the chosen source] … know about “where earthquakes come from”?

☐ 1 ☐ 2 ☐ 3 ☐ 4 ☐ 5 ☐ 6 ☐ 7 ☐ Don’t know

2. How much do you trust … [the chosen source’s] … response when asking “where earthquakes come from”?

☐ 1 ☐ 2 ☐ 3 ☐ 4 ☐ 5 ☐ 6 ☐ 7 ☐ Don’t know

3. So, if you had 3 hours to learn “where earthquakes come from” in order to prepare a Natural Sciences presentation, you would do it by learning with … [the chosen source] … . How much do you expect to learn about “where earthquakes come from” this way?

☐ 1 ☐ 2 ☐ 3 ☐ 4 ☐ 5 ☐ 6 ☐ 7 ☐ Don’t know

4. How much do you expect to enjoy learning about “where earthquakes come from” this way?

☐ 1 ☐ 2 ☐ 3 ☐ 4 ☐ 5 ☐ 6 ☐ 7 ☐ Don’t know

5. And if you got stuck during the learning process and can’t move forward, what would you do?

- ☐ Take a Break
- ☐ Keep trying
- ☐ Person (specify which one)
- ☐ Platform (specify which one)
- ☐ Nothing
- ☐ Give up
- ☐ Other
- ☐ Don’t know → Prompt once: “That’s okay, but… think about it, why?”

ID:

6. Only for participants who selected scores <= 3 in (3) and/or (4): Why did you choose to learn with … [the chosen source] … if you think you would learn little / enjoy it little?

- ☐ Couldn't think of another option
- ☐ Ease
- ☐ Time
- ☐ Motivation (enjoyment)
- ☐ Expertise (source's preparation)
- ☐ Genius (because the source I chose is a genius)
- ☐ Presentations require working independently (e.g.: when it's a presentation you have to look it up yourself, not ask the teacher)
- ☐ Influence of another person (e.g.: my mom tells me to read more books)
- ☐ Comparison with another option
- ☐ Other
- ☐ Tautological → Prompt once
- ☐ Don't know → Prompt once: "That's okay, but… think about it, why?"

End time:
